# Olfactory ensheathing cells exosomes enhance neurological recovery in brain‐injured rats by modulating Nrf2‐ferroptosis pathway

**DOI:** 10.1002/btm2.70097

**Published:** 2025-11-29

**Authors:** Xin‐li Chen, Yi‐bin Liu, Cheng‐ye Lin, Shu Lin, He‐fan He, Wei‐feng Liu

**Affiliations:** ^1^ Department of Anesthesiology The Second Affiliated Hospital of Fujian Medical University Quanzhou Fujian China; ^2^ Department of Orthopedics The Second Affiliated Hospital of Fujian Medical University Quanzhou Fujian China; ^3^ Group of Neuroendocrinology Garvan Institute of Medical Research Sydney New South Wales Australia; ^4^ Centre of Neurological and Metabolic Research The Second Affiliated Hospital of Fujian Medical University Quanzhou Fujian China

**Keywords:** exosomes, ferroptosis, Nrf2 pathway, olfactory ensheathing cells, traumatic brain injury

## Abstract

This study aimed to evaluate the therapeutic potential of the olfactory ensheathing cells (OECs) exosomes (EXOs) in traumatic brain injury (TBI) and the regulatory role of nuclear factor E2‐related factor (Nrf2). Rats were divided into Sham, TBI, OECs‐EXOs (EXO), and OECs‐EXOs plus Nrf2 inhibitor (ML385) groups. Neurological function was assessed using the modified neurological severity score, the Morris water maze (MWM), and the Barnes maze tests. Brain injury, Fe^2+^ accumulation, and mitochondrial damage were evaluated using histopathological imaging and ELISA kits. Expression levels of Nrf2 and ferroptosis‐related proteins were analyzed using western blot and qPCR. TBI rats exhibited significant neurological dysfunction, elevated serum injury markers and inflammatory cytokines, increased brain Fe^2+^ and malondialdehyde (MDA), and altered expression of ferroptosis‐related proteins compared with Sham rats. The ML385 group exhibited reduced Nrf2 expression and attenuated OECs‐EXOs‐mediated therapeutic effects, suggesting a critical role of Nrf2 in the efficacy of OECs‐EXOs. Overall, OECs‐EXOs attenuated TBI‐induced neuroinflammation and oxidative stress and promoted neuronal repair and neurological recovery, likely via Nrf2‐regulated ferroptosis‐related pathways.

## Graphical abstract

This study demonstrates that olfactory ensheathing cell‐derived exosomes (OEC‐EXOs) improve neurological outcomes in traumatic brain injury (TBI) by reducing neuroinflammation, oxidative stress, and ferroptosis through Nrf2 activation, highlighting their potential as a novel therapeutic strategy for TBI management.

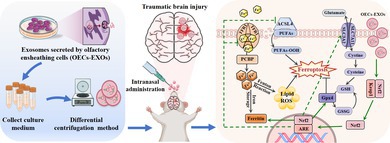

**Translational Impact Statement**

This study demonstrates that olfactory ensheathing cells‐derived exosomes (OECs‐EXOs) enhance neurological recovery and reduce brain injury in traumatic brain injury (TBI) by modulating oxidative stress and ferroptosis via the Nrf2 pathway, highlighting their potential as a novel therapeutic strategy for TBI.

## 1 INTRODUCTION

Traumatic brain injury (TBI) is a severe form of neurotrauma with limited effective treatment options. TBI is a leading cause of post‐traumatic disability and death, with mortality rates of 37% in Europe and 30.5% in the United States.^1^, ^2^ Recent studies indicate that ferroptosis is closely associated with TBI pathogenesis. Transplantation of olfactory ensheathing cells (OECs) is expected to alleviate cognitive dysfunction by modulating the nuclear factor E2‐related factor 2 (Nrf2) signaling pathway, which is crucial for ferroptosis regulation. However, the risks associated with direct cell‐based therapies have prompted the exploration of cell‐derived exosomes as novel therapeutic strategies for TBI. TBI results in a primary injury that causes structural brain abnormalities and intracerebral hemorrhage, followed by a secondary injury characterized by cell death, edema, oxidative stress, iron accumulation, endoplasmic reticulum stress, inflammation, and immune responses.^3^, ^4^ These pathological processes interact synergistically with each other, resulting in sustained neuronal cell death and further complicating TBI treatment.^1^ Therefore, effective treatments are urgently needed to restore neurological function and improve long‐term prognosis after brain injury.

Ferroptosis is a novel form of regulated cell death characterized by disturbed iron metabolism and redox imbalance, and it plays a significant role in various neurological disorders, including TBI.^4^, ^5^ Hu et al. revealed characterized ferroptotic pathology in TBI, including iron accumulation, dysregulated iron metabolism, upregulation of iron‐handling genes, reduced glutathione (GSH) peroxidase activity, elevated lipid reactive oxygen species (ROS), and mitochondrial damage.^6^ Ferroptosis is regulated by protective pathways such as the glutathione peroxidase 4 (Gpx4) axis, in which Gpx4 functions as an antioxidant enzyme that limits ROS accumulation and suppresses ferroptosis.^7^ Ferroptosis driven by iron‐dependent lipid peroxidation is closely associated with stress; therefore, it is essential to assess the levels of lipid peroxidation–related biomarkers.^8^ Nrf2, a transcription factor that initiates endogenous antioxidant responses, serves as a negative regulator of ferroptosis.^9^ Under stress conditions, Nrf2 translocates to the nucleus, activating the transcription of antioxidant enzymes such as heme oxygenase 1 and Gpx4.^10^ Nrf2 also promotes GSH synthesis and reduces cellular susceptibility to ferroptosis.^11^ Gpx4 is a key downstream ferroptosis target regulated by Nrf2 and is associated with solute carrier family 7 member 11 (SLC7A11).^12^ Gpx4 mediates ferroptosis by facilitating cysteine uptake via SLC7A11, which is also regulated by Nrf2. These findings suggest that Nrf2 is a potential therapeutic target for TBI.^13^

Recent advances have highlighted the use of cell‐based therapies and cell‐derived products for nerve injury treatment.^14^ OECs have shown potential for TBI treatment because of their ability to proliferate, cross glial scars, and survive within the central nervous system.^15^ OECs secrete adhesion molecules, extracellular matrix components, and neurotrophic factors, and they modulate immune and inflammatory responses, thereby improving motor function in TBI rats.^16^ OECs promote neural repair even in spinal cord transection models.^17^ OECs are more readily obtainable than stem cells, and autologous transplantation has demonstrated therapeutic efficacy in clinical studies.^18^ While OECs exhibit promising neural repair effects, their transplantation is not without risks. These risks include tumorigenesis, thrombosis, immune rejection, and abnormal mass formation at graft sites, all of which have been documented in previous studies.^19^, ^20^ These complications underscore the need for safer alternatives, such as OECs‐derived exosomes (OECs‐EXOs), which may offer therapeutic benefits without the risks associated with direct cellular transplantation.^21^

Exosomes contain bioactive cargo, including proteins, lipids, DNA, and RNA, which can be delivered to recipient cells.^22^ Mesenchymal stem cell‐derived exosomes modulate astrocyte and microglial activation and neuroinflammation in TBI.^23^ In addition, neuron‐derived exosomes from induced pluripotent stem cells promote neurogenesis and neuronal circuitry development following TBI.^24^ OECs‐EXOs have shown the potential to modulate microglia/macrophage polarization, thereby conferring neuroprotection after spinal cord injury.^25^ Given their similar pathogenesis, OECs‐EXOs may have therapeutic potential for TBI. Ailing et al. demonstrated that OECs might inhibit neuroinflammation and oxidative stress in brain injury through activation of the Nrf2 pathway.^26^ Nevertheless, the role of OECs‐EXOs in regulating ferroptosis via the Nrf2 pathway, thereby improving neurological recovery after TBI, remains unknown. This study aimed to investigate the protective effects of OECs‐EXOs in TBI rats and elucidate the underlying mechanisms. Oxidative stress indicators and ferroptosis markers were assessed to investigate the role of OECs‐EXOs in modulating Nrf2‐mediated regulation of ferroptosis and their therapeutic effects in TBI.

## 2 RESULTS

### 2.1 Characterization and identification of OECs

**FIGURE 1:**
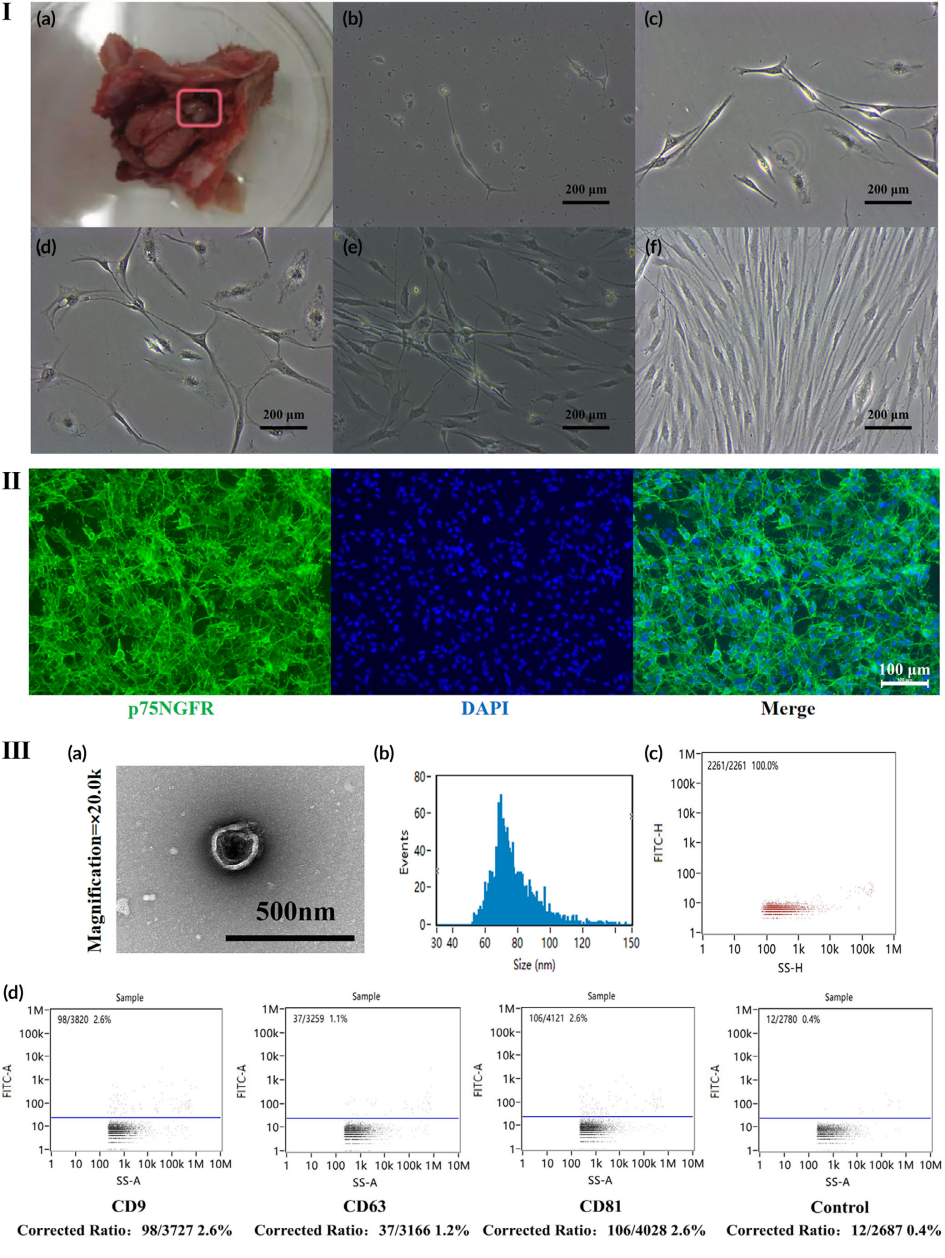
Culture, identification, and characterization of olfactory ensheathing cells (OECs) and OECs‐derived exosomes (OECs‐EXOs). (I) Primary culture of OECs: (a) Olfactory bulb tissue was collected from euthanized rats and OECs were isolated. (b) On Day 1, OECs adhered to the culture dish after purification by differential adhesion. (c) Marked cell proliferation was observed on Day 2. (d) By Day 3, cells displayed a multipolar morphology. (e) From Day 5 onward, cells formed an interconnected network. (f) By Day 10, cells showed fenestrated growth with >80% confluence. (II) Immunofluorescent identification of OECs by p75 nerve growth factor receptor (p75NGFR). Green: p75NGFR; blue: DAPI. Magnification: 100×. (III) Characterization of OECs‐EXOs: (a) Transmission electron microscopy (TEM) images showing approximately spherical vesicles with a visible bilayer membrane at ×20.0k magnification. (b) Particle size distribution of OECs‐EXOs. (c) Concentration profile of OECs‐EXOs. (d) Flow cytometry analysis confirming positive expression of exosomal markers CD9, CD63, and CD81.

After 1–2 days of differential adhesion purification, OECs exhibited a bipolar morphology and began to proliferate when observed under light microscopy (Figure 1(Ia–c)). During Days 3–5, the cells proliferated rapidly and formed a reticulated network (Figure 1(Id,e)). By Days 7–10, the cells displayed a palisade‐like arrangement and reached confluence, at which point they were ready for passaging (Figure 1(If)). p75NGFR immunofluorescence staining was used to identify OECs, with green fluorescence observed on the cell membrane and blue fluorescence in the nuclei, confirming their identity as OECs (Figure 1(II)).

### 2.2 Identification of exosomes from OECs

Representative transmission electron microscopy (TEM) image showed that exosomes were approximately spherical vesicles with a clearly defined bilayer membrane (Figure 1(IIIa)). Nanoparticle tracking analysis revealed particle diameters ranging from 30 to 150 nm, with an average particle size of 77.23 nm and a concentration of 6.15 × 10^9^ particles/mL (Figure 1(IIIb,c)). High‐sensitivity flow cytometry demonstrated the positive expression of the exosomal markers CD9, CD63, and CD81 in the exosomes group compared with the control (Figure 1(IIId)). These data suggest the isolated OECs‐EXOs possess typical exosome characteristics.^27^

### 2.3 Localization of OECs‐EXOs in vivo and in brain tissue

#### 2.3.1 Fluorescence imaging of DiR‐labeled OECs‐EXOs in vivo

**FIGURE 2:**
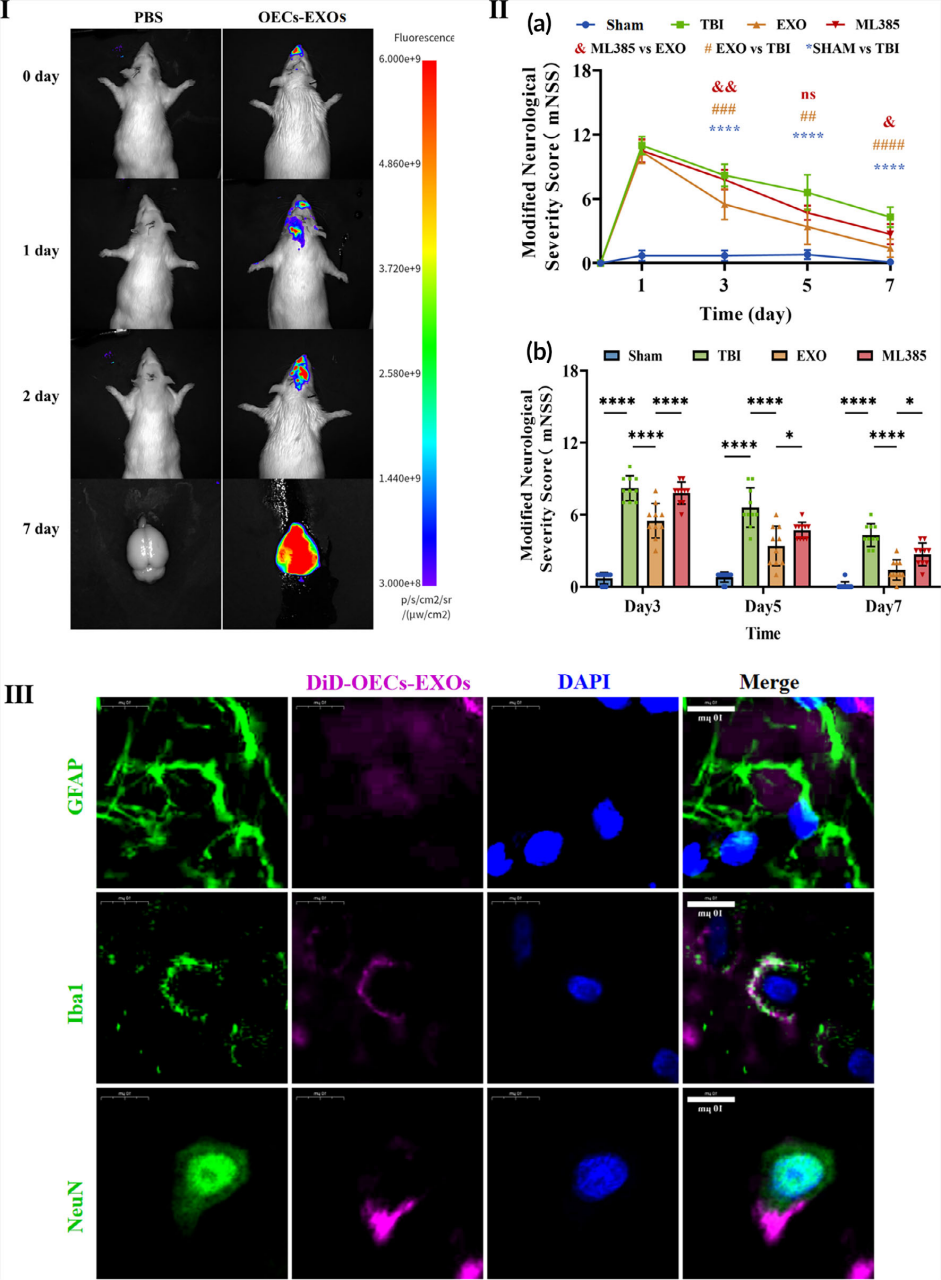
Distribution of OECs‐EXOs and neurological functional recovery. (I) Fluorescence imaging of rat heads and brains on Days 0, 1, 2, and 7 after intranasal administration of PBS or DiR‐labeled OECs‐EXOs. (II) Neurological function assessment: (a) Time course of modified neurologic severity score (mNSS) from Day 1 to Day 7 post‐TBI. (b) Comparison of mNSS among groups on Days 3, 5, and 7 (* and # indicate statistical significance vs. the TBI group; & indicates statistical significance vs. the ML385 group). (III) Immunofluorescence co‐localization of DiD‐labeled OECs‐EXOs with NeuN (neurons), GFAP (astrocytes), and Iba1 (microglia) in brain sections. Statistics for (II): (a) Two‐way repeated‐measures ANOVA; (b) two‐way ANOVA. All data passed normality testing. *n* = 10 per group. *p < 0.05, **p < 0.01, ***p < 0.001, ****p < 0.0001.

Fluorescence imaging using DiR‐labeled OECs‐EXOs showed that the signal gradually migrated from the nasal cavity toward the brain injury site after OECs‐EXOs treatment. On Day 7, dissection of the brain tissue revealed concentrated fluorescent signals in the injured region, suggesting that certain neural cells may have absorbed OECs‐EXOs and subsequently migrated to the injury site (Figure 2(I)).

#### 2.3.2 Immunofluorescence co‐localization of DiD‐labeled OECs‐EXOs in brain tissue sections

To further investigate the cellular uptake of DiD‐labeled OECs‐EXOs after intranasal delivery, we performed immunofluorescence staining for neuronal (NeuN), astrocytic (GFAP), and microglial (Iba1) markers in brain tissue sections. Co‐localization analysis revealed that DiD‐labeled OECs‐EXOs were present in neurons, astrocytes, and microglia (Figure 2(III)). These results suggest that OECs‐EXOs are taken up by multiple cell types in the brain following intranasal delivery.

### 2.4 Effect of OECs‐EXOs on improving neurological function in TBI rats

All groups exhibited varying degrees of neurological recovery within 1 week post‐injury (Figure 2(IIa)). On Day 1, the Sham group showed normal neurological function, whereas the other groups displayed comparable moderate injury scores without significant differences. On Days 3, 5, and 7, the OECs‐EXOs‐treated group demonstrated significantly lower modified neurological severity score (mNSS) scores than the TBI group (*p* < 0.05), indicating improved neurological recovery. However, this improvement was markedly attenuated following ML385 administration (Figure 2(IIb); *p* < 0.05).

### 2.5 Spatial learning and memory

#### 2.5.1 Morris water maze

**FIGURE 3:**
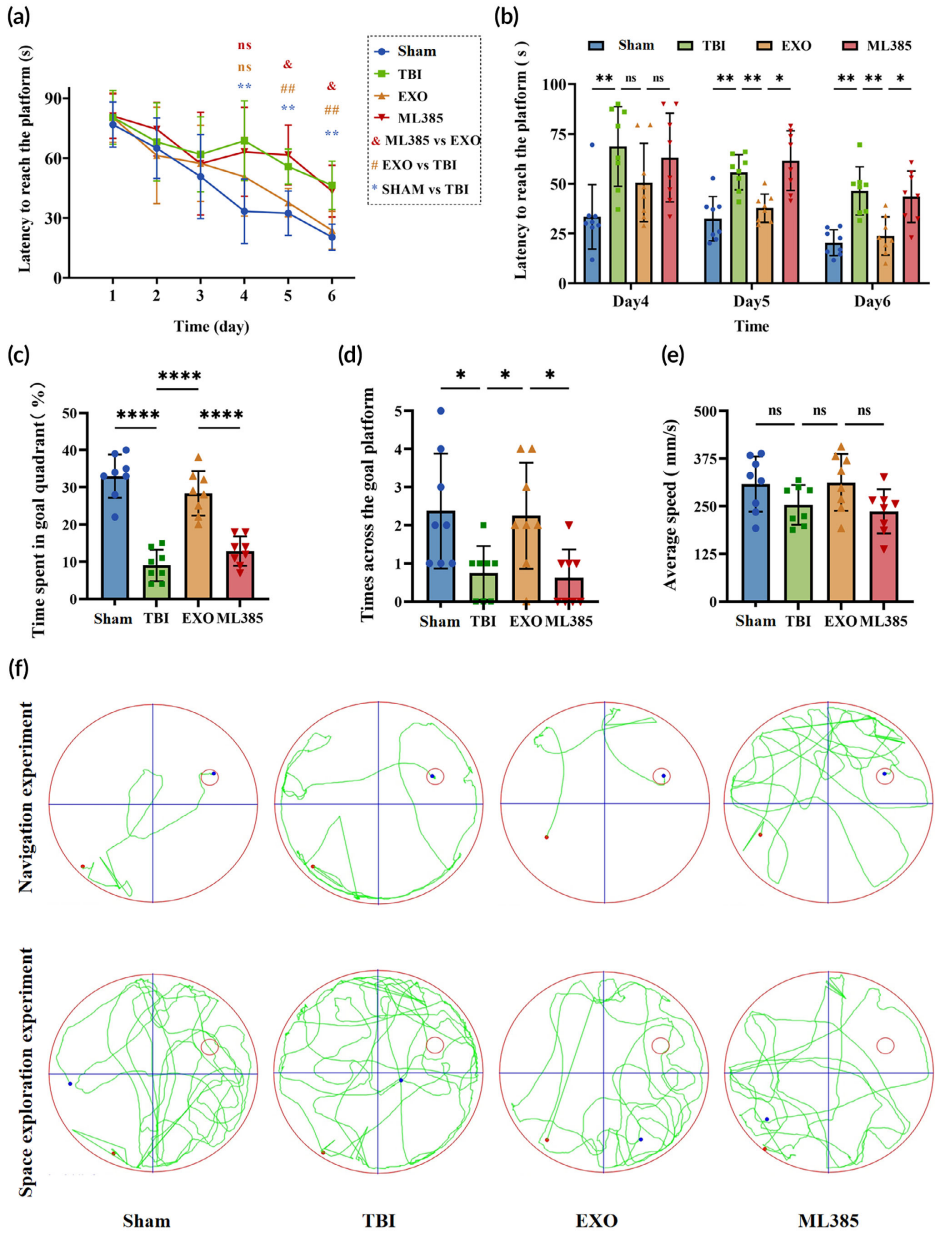
Morris water maze performance 1–7 days after TBI. (a) Changes in escape latency during acquisition training from Day 1 to Day 6. (b) Escape latency on training days 4–6. (c) Percentage of time spent in the target quadrant in the Day 7 probe trial. (d) Number of platform crossings in the Day 7 probe trial. (e) Average swimming speed in the Day 7 probe trial. (f) Representative swimming paths during orientation navigation and in the probe test. Statistics for this figure: (a) Two‐way repeated‐measures ANOVA; (b) two‐way ANOVA; (c–e) one‐way ANOVA. All data passed normality testing. *n* = 8 per group. *p < 0.05, **p < 0.01, ***p < 0.001, ****p < 0.0001.

From Day 1 to Day 6, escape latencies progressively decreased, indicating the acquisition of spatial learning and memory (Figure 3a; *p* < 0.0001). On Days 5 and 6, escape latencies were longer in the TBI group than in the Sham group. However, the OECs‐EXOs treatment shortened escape latencies, although this effect was attenuated by ML385 co‐administration (Figure 3b; *p* < 0.05). In the probe trial, the Sham group spent more time in the target quadrant than the TBI group, and the EXO group spent more time in the target quadrant than the TBI and ML 385 groups (Figure 3c; *p* < 0.0001). The number of platform crossings was higher in the Sham group compared to that in the TBI group, and platform crossings in the EXO group were higher than both the TBI and ML385 groups (Figure 3d; *p* < 0.05). No significant differences were observed in swimming speed among the groups, suggesting that the observed effects reflected differences in learning and memory rather than motor function (Figure 3e; *p* > 0.05). The typical trajectories on Day 7 of the navigation experiment revealed distinct search patterns: location‐to‐goal for Sham, circular for TBI, goal‐directed for EXO, and random for ML385 (Figure 3f). In the space exploration experiment, the Sham and EXO groups spent more time in the target quadrant, while the TBI and ML385 groups spent more time in the non‐target areas (Figure 3f). These findings suggest that OECs‐EXOs treatment improved cognitive function, although this effect was partially attenuated by co‐administration of ML385.

#### 2.5.2 Barnes maze

**FIGURE 4:**
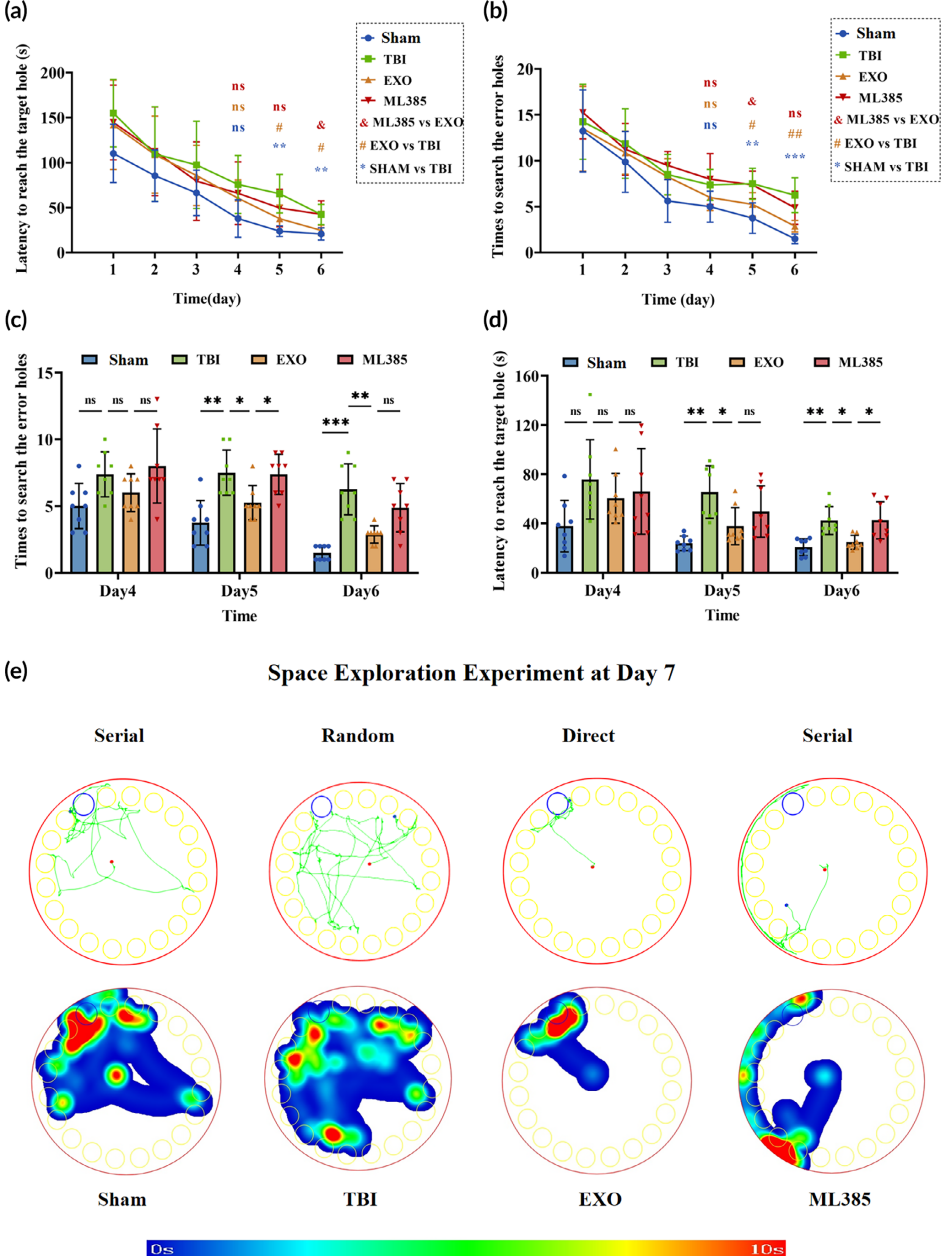
Barnes maze performance 1–7 days after TBI. (a) Number of errors during hole exploration across training days 1–6. (b) Number of errors on training days 4–6. (c) Escape latency across training days 1–6. (d) Escape latency on training days 4–6. (e) Representative trajectories (top row) and heat maps of time spent in each quadrant (bottom row) in the spatial exploration test. Statistics for this figure: (a, b) Two‐way repeated‐measures ANOVA; (c, d) two‐way ANOVA. All data passed normality testing. *n* = 8 per group. *p < 0.05, **p < 0.01, ***p < 0.001, ****p < 0.0001.

Training revealed significant reductions in exploration errors over time, indicating improved spatial memory (Figure 4a; *p* < 0.001). On Days 5 and 6, the Sham and EXO groups made fewer errors than the TBI group, with a significant difference between the EXO and ML385 groups on Day 5 (Figure 4b; *p* < 0.05). The latency to enter the target box decreased over time, reflecting the formation of spatial memory (Figure 4c; *p* < 0.0001). On Days 5 and 6, latencies were longer in the TBI group than in the Sham and EXO groups, with a significant difference between the EXO and ML385 groups on Day 6 (Figure 4d; *p* < 0.05). Heat maps on Day 7 showed different search patterns: continuous for Sham and ML385, spatial for EXO, and random for TBI (Figure 4e). These results suggest that OECs‐EXOs treatment improved cognitive function post‐TBI, whereas ML385 partially attenuated this effect.

### 2.6 OECs‐EXOs alleviate brain and neuronal damage in TBI rats

#### 2.6.1 H&E and Nissl staining

**FIGURE 5:**
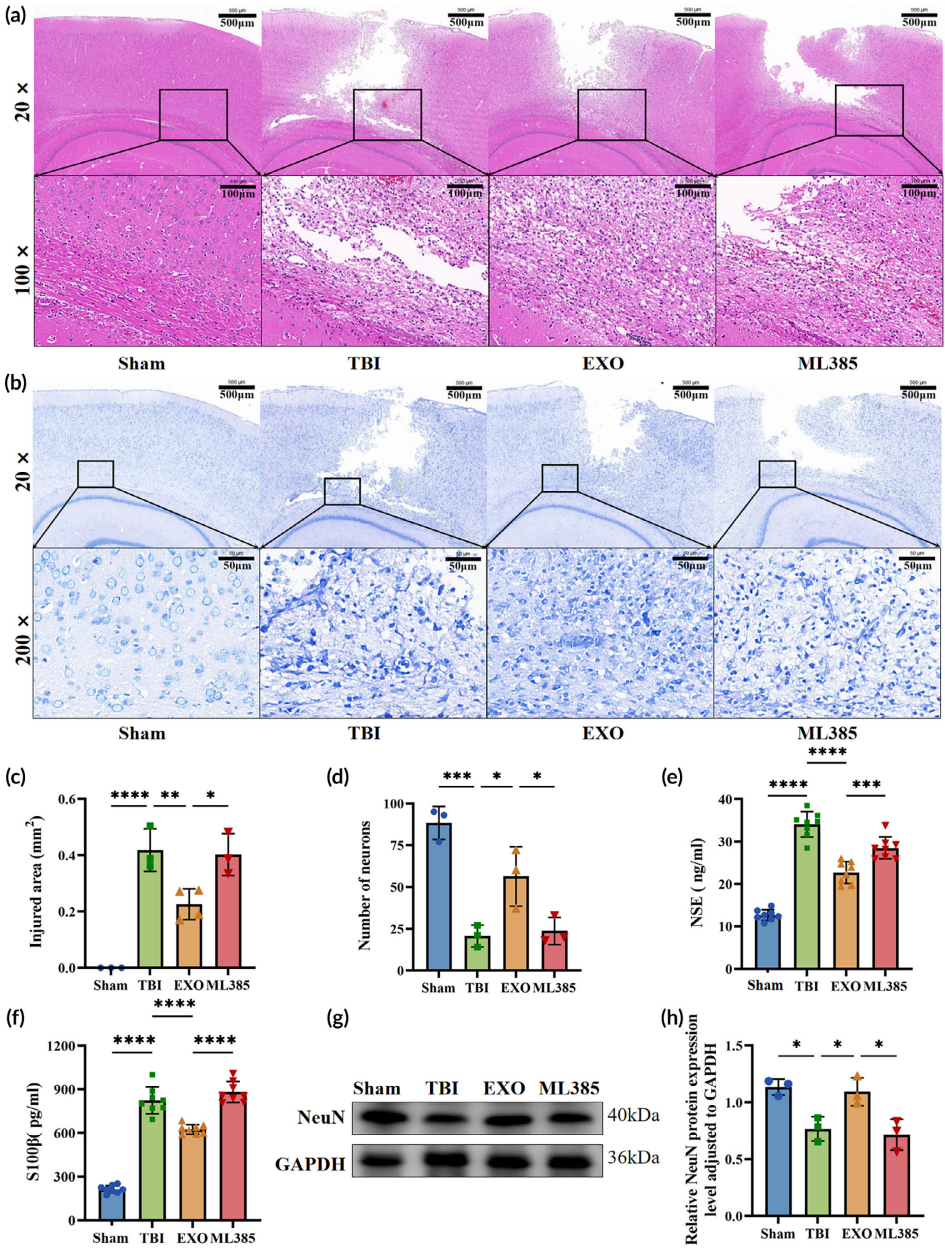
Histological and biochemical assessment of brain injury. (a) Hematoxylin and eosin (H&E) staining of brain sections from each group. (b) Nissl staining of brain sections from each group. (c) Quantification of damaged cortical area based on H&E staining. (d) Neuronal counts in the injured region based on Nissl staining. (e) Serum neuron‐specific enolase (NSE) levels on Day 7 post‐injury. (f) Serum S100β levels on Day 7 post‐injury. (g, h) Immunofluorescent staining and quantification of NeuN expression in brain tissue. Statistics for this figure: (c–f, h) One‐way ANOVA. All data passed normality testing. (c, d, h) *n* = 3 per group; (e, f) *n* = 8 per group. *p < 0.05, **p < 0.01, ***p < 0.001, ****p < 0.0001.

H&E staining (Figure 5a) revealed intact brain tissue in the Sham group, whereas the TBI group exhibited necrosis, tissue loss, and structural disruption. Lesion area analysis showed significantly smaller areas of damage in the EXO group than in the TBI and ML385 groups (Figure 5c; *p* < 0.05), suggesting that OECs‐EXOs treatment reduced brain damage. However, this effect was diminished by the ML385 group. Nissl staining (Figure 5b) revealed normal neurons in the Sham group, whereas the TBI group had fewer neurons with altered morphology, nuclear pyknosis, and indistinct Nissl bodies. Neuronal counts were significantly higher in the EXO group than in the TBI and ML385 groups (Figure 5d; *p* < 0.05).

#### 2.6.2 Biomarkers of brain and neuronal injury

NSE and S100β levels were elevated in the TBI group but significantly reduced after OECs‐EXOs treatment (Figure 5e,f; *p* < 0.0001). Post‐treatment, these markers were significantly lower in the EXO group than in the TBI group (*p* < 0.0001); however, their levels were elevated when ML385 was co‐administered with OECs‐EXOs (*p* < 0.001). The expression of NeuN (a marker of mature neurons) was also assessed in brain tissues (Figure 5g,h). NeuN expression was higher in the Sham and EXO groups than in the TBI group (*p* < 0.05) but was lower in the ML385 group than in the EXO group (*p* < 0.05). These results suggest that OECs‐EXOs treatment promotes neuronal repair and reduces neuronal damage, though ML385 may impair these effects.

#### 2.6.3 Expression of cell death–related genes

**FIGURE 6:**
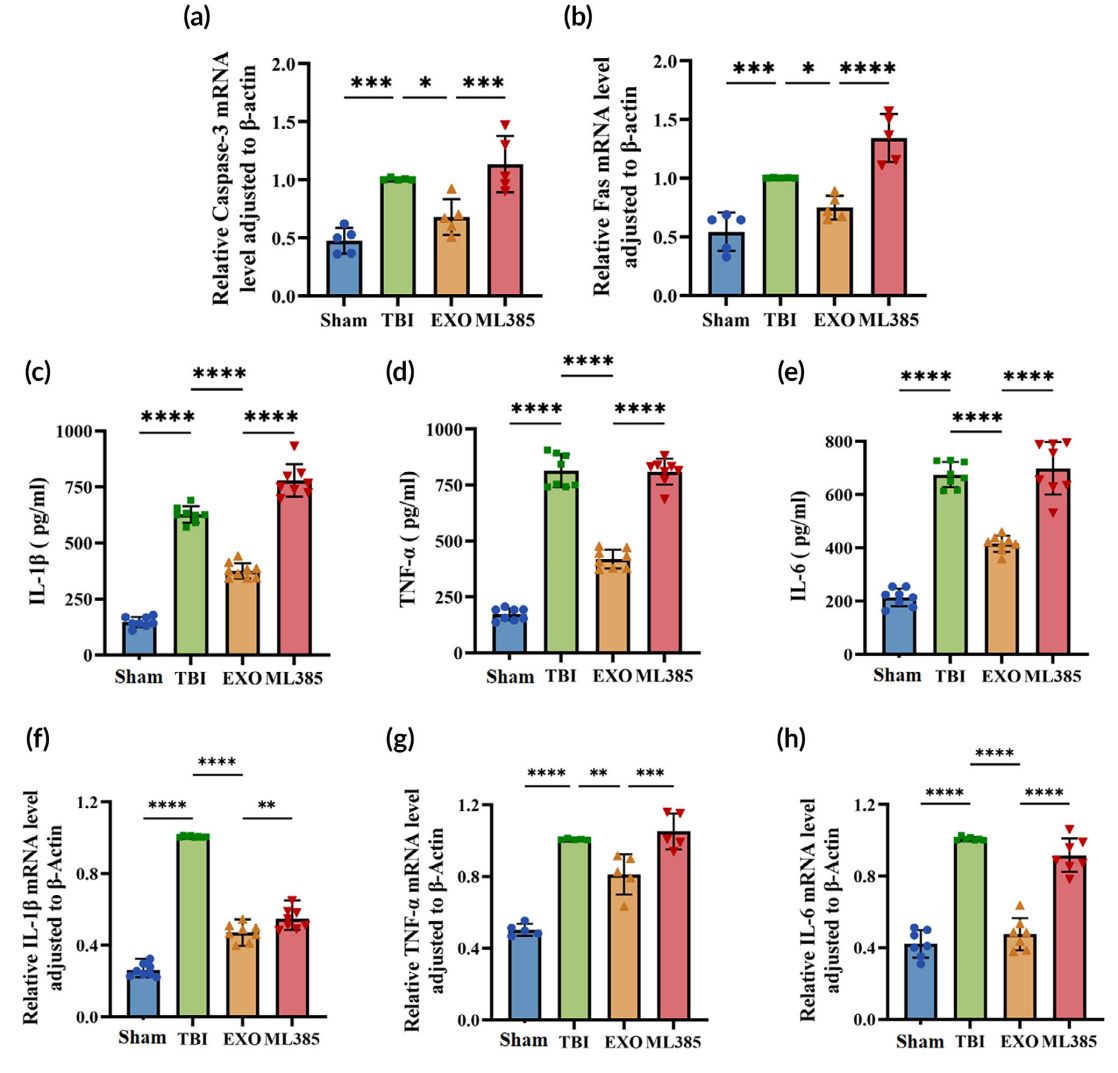
Apoptosis and inflammatory responses on Day 7 post‐TBI. (a, b) Caspase‐3 and Fas mRNA expression levels in brain tissue. (c–e) Serum levels of IL‐1β, TNF‐α, and IL‐6. (f–h) IL‐1β, TNF‐α, and IL‐6 mRNA expression levels in brain tissue. Statistics for for this figure: (a–h) One‐way ANOVA. All data passed normality testing. (a, b, g) *n* = 5 per group; (c–f) *n* = 8 per group; (h) *n* = 7 per group. *p < 0.05, **p < 0.01, ***p < 0.001, ****p < 0.0001.

Compared with the Sham group, the TBI group showed a marked increase in caspase‐3 (cysteine‐aspartic protease 3) and Fas (fatty acid synthase) mRNA levels, indicating activation of cell death pathways after injury. OECs‐EXOs treatment significantly reduced the TBI‐induced upregulation of both genes (*p* < 0.05 vs. TBI), whereas co‐administration of ML385 partially reversed these effects, with higher caspase‐3 and Fas expression in the ML385 group than in the EXO group (Figure 6a,b; *p* < 0.05). These results further support the notion that OECs‐EXOs treatment attenuates neuronal damage in TBI rats and that this protective effect is at least partly dependent on Nrf2.

### 2.7 OECs‐EXOs reduce serum and brain tissue inflammatory gene expression

IL‐1β levels were significantly higher in serum and brain tissues in the TBI group than in the Sham group (Figure 6c,f; *p* < 0.0001). OECs‐EXOs treatment significantly reduced IL‐1β levels relative to the TBI group (*p* < 0.0001), although IL‐1 β levels remained higher in the ML385 group than in the EXO group (*p* < 0.01). TNF‐α levels were higher in the TBI group than in the Sham group (Figure 6d,g; *p* < 0.0001), significantly lower in the EXO group post‐treatment (*p* < 0.01), and elevated again in the ML385 group than in the EXO group (*p* < 0.001). Similarly, IL‐6 levels in both serum and brain tissue were higher in the TBI group than in the Sham and EXO groups and were higher in the ML385 group than in the EXO group (Figure 6e,h; *p* < 0.0001). These findings suggest that OECs‐EXOs treatment improves neural function and promotes neuronal repair in TBI by reducing inflammation, an effect that may be mediated by Nrf2.

### 2.8 OECs‐EXOs reduce oxidative stress, Fe^2+^ accumulation, and mitochondrial damage after TBI

#### 2.8.1 Oxidative stress reduction

**FIGURE 7:**
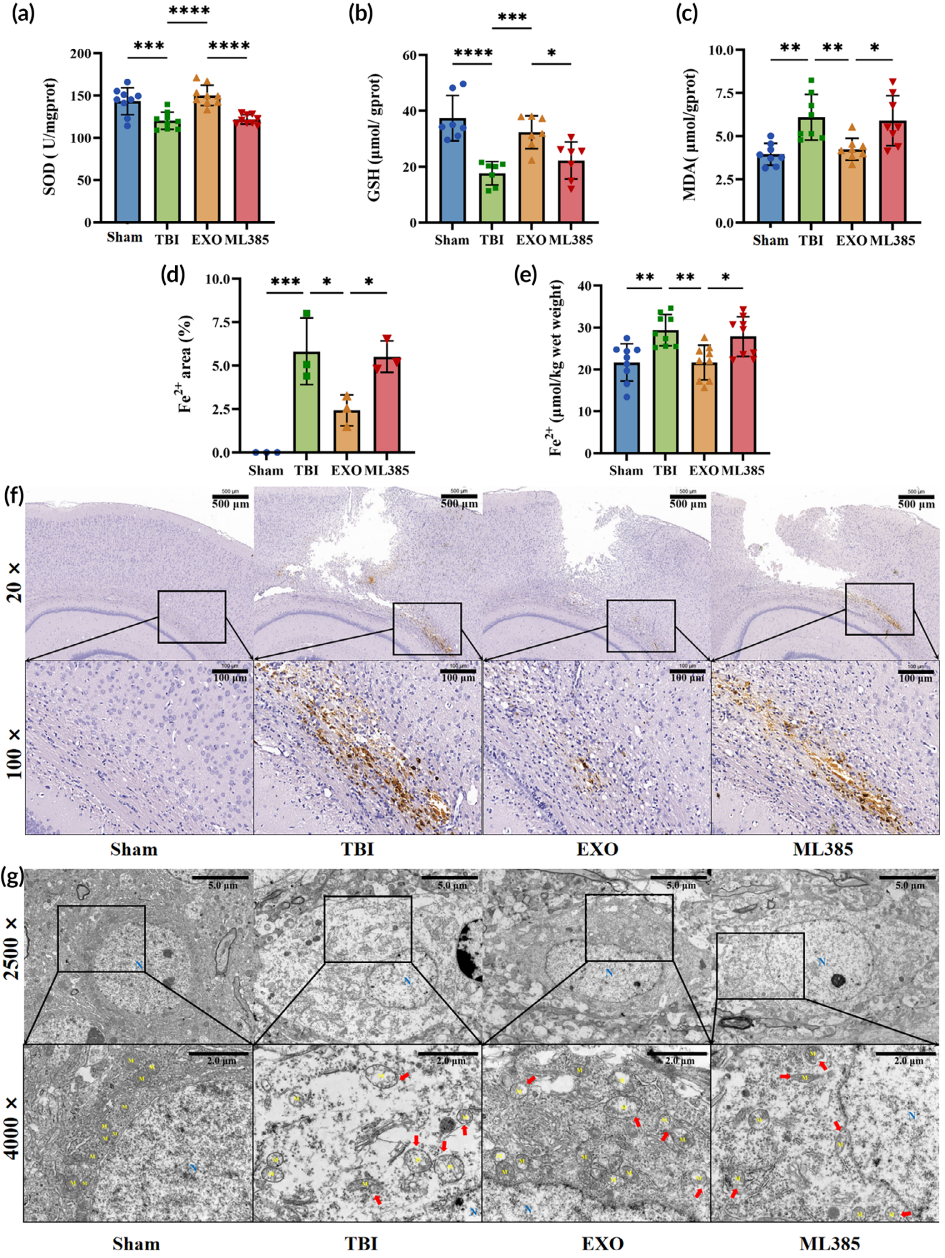
Oxidative stress and iron deposition in brain tissue. (a) Superoxide dismutase (SOD) activity. (b) Glutathione (GSH) levels. (c) Malondialdehyde (MDA) levels. (d) Fe^2+^‐positive area by Prussian blue staining. (e) Fe^2+^ content in brain tissue. (f) Representative Prussian blue‐stained sections. (g) Transmission electron microscopy of mitochondria (N, nucleus; Mi, mitochondria; red arrows, damaged mitochondria). Statistics for this figure: (a–e) One‐way ANOVA. All data passed normality testing. (a, e) *n* = 9 per group; (b) *n* = 7 per group; (c) *n* = 8 per group; (d) *n* = 3 per group. *p < 0.05, **p < 0.01, ***p < 0.001, ****p < 0.0001.

As shown in Figure 7a–c, the TBI group exhibited higher MDA levels (*p* < 0.01), lower GSH levels (*p* < 0.0001), and reduced SOD activity (*p* < 0.001) than the Sham group, indicating oxidative stress. OECs‐EXOs treatment decreased MDA (*p* < 0.01), increased GSH (*p* < 0.001), and enhanced SOD activity (*p* < 0.0001), whereas ML385 co‐administration partially reversed these effects (*p* < 0.05). These findings suggest that OECs‐EXOs treatment reduces lipid peroxidation and oxidative stress in TBI via Nrf2.

#### 2.8.2 Fe^2+^ accumulation

Fe^2+^ was not detected in the Sham group, and there were significantly fewer Fe^2+^‐positive areas in the EXO group than in the TBI and ML385 groups (Figure 7d,f; *p* < 0.05). Fe^2+^ content analysis showed that the TBI group had higher Fe^2+^ levels than the Sham group (Figure 7e; *p* < 0.01), whereas the EXO group had significantly lower Fe^2+^ levels than the TBI and ML385 groups (*p* < 0.05). These results suggest that OECs‐EXOs treatment reduces Fe^2+^ accumulation after TBI and that Nrf2 inhibition by ML385 reverses this effect.

#### 2.8.3 Mitochondrial damage

In the TBI and ML385 groups, a reduced mitochondrial number, membrane rupture, loss of cristae, and partial shrinkage were observed, which were consistent with mitochondrial damage induced by ferroptosis^4^ (Figure 7g). Conversely, the EXO group exhibited an improved mitochondrial number and structure compared with the TBI group, suggesting that OECs‐EXOs treatment mitigates TBI‐induced mitochondrial injury.

### 2.9 OECs‐EXOs modulate the ferroptosis‐related molecular levels in brain tissue after TBI

**FIGURE 8:**
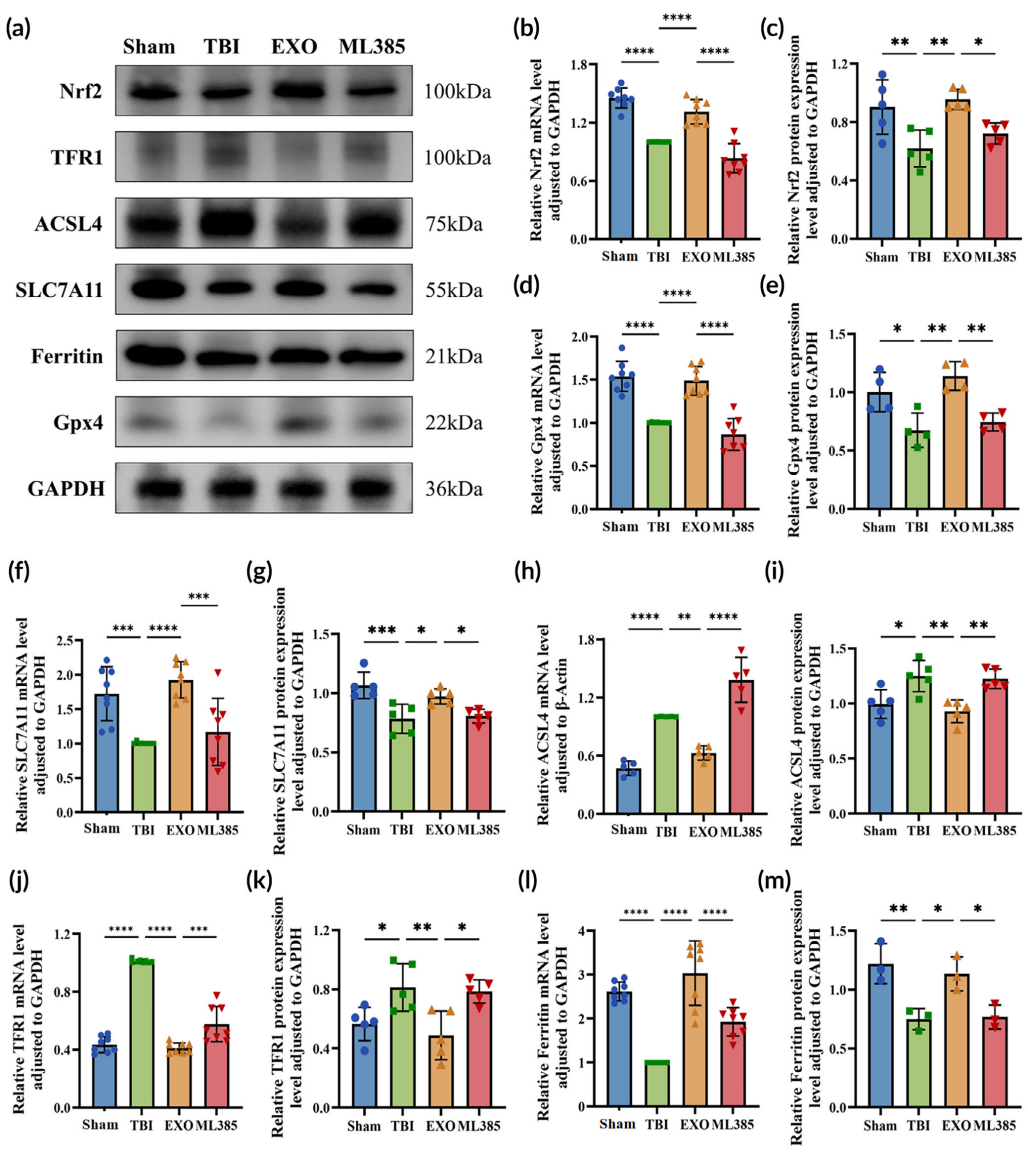
Expression of ferroptosis‐related molecules in rat brain on Day 7 post‐TBI. (a) Representative western blots of Nrf2, TFR1, ACSL4, SLC7A11, ferritin, and GPX4. (b, c) Nrf2 mRNA and protein expression. (d, e) GPX4 mRNA and protein expression. (f, g) SLC7A11 mRNA and protein expression. (h, i) ACSL4 mRNA and protein expression. (j, k) TFR1 mRNA and protein expression. (l, m) Ferritin mRNA and protein expression. Nrf2, nuclear factor E2‐related factor 2; GPX4, glutathione peroxidase 4; SLC7A11, solute carrier family 7 member 11; ACSL4, acyl‐CoA synthetase long‐chain family member 4; TFR1, transferrin receptor 1. Statistics for this figure: (b–m) One‐way ANOVA. All data passed normality testing. (b, d, f, j, l) *n* = 8 per group; (c, g, h, i, k) *n* = 5 per group; (e) *n* = 4 per group; (m) *n* = 3 per group. *p < 0.05, **p < 0.01, ***p < 0.001, ****p < 0.0001.

Nrf2 levels were significantly lower in the TBI group than in the Sham group (*p* < 0.01), whereas Nrf2 levels were significantly higher in the EXO group than in the TBI group (*p* < 0.01; Figure 8). After ML385 treatment, Nrf2 expression was significantly downregulated compared with the EXO group (*p* < 0.05). These results suggest that Nrf2 levels decrease after TBI and that OECs‐EXOs treatment effectively increased Nrf2 expression, whereas ML385 reduced this effect. Ferroptosis markers were evaluated in brain tissue, including the pro‐ferroptotic proteins TFR1 and ACSL4 and the anti‐ferroptotic regulators ferritin, Gpx4, and SLC7A11 (Figure 8).^8^ TFR1 and ACSL4 were significantly upregulated in the TBI group relative to the Sham group (*p* < 0.05), whereas ferritin, Gpx4, and SLC7A11 levels were significantly reduced (*p* < 0.05). In the EXO group, TFR1 and ACSL4 levels were significantly lower than in the TBI group (*p* < 0.01), and ferritin, Gpx4, and SLC7A11 levels were significantly upregulated (*p* < 0.05), suggesting that OECs‐EXOs treatment may inhibit or attenuate ferroptosis. However, ML385 treatment reduced these effects (*p* < 0.05), suggesting that the therapeutic effects of OECs‐EXOs on TBI may involve Nrf2‐dependent modulation of ferroptosis‐related pathways.

## 3 DISCUSSION

OECs promote neurorecovery in TBI through mechanisms such as phagocytosis, secretion, and immunomodulation.^16^ However, the risks of tumorigenesis, thrombosis, and immune rejection associated with cell‐based in situ therapy make cell‐derived EXOs a promising alternative approach for TBI treatment.^28^ OECs improve cognitive deficits after TBI by modulating the Nrf2 pathway,^26^ which plays a key role in limiting oxidative stress and ferroptosis in TBI.^29^ The primary findings of this study are as follows: OECs‐EXOs improved TBI‐induced neurological deficits and cognitive dysfunction and reduced lesion area, neuronal loss, brain tissue damage, and neuroinflammatory factor levels; OECs‐EXOs attenuated oxidative stress, iron metabolism disorders, and mitochondrial damage; inhibition of Nrf2 expression eliminated OECs‐EXOs' therapeutic effects; the therapeutic mechanism of OECs‐EXOs may involve regulating ferroptosis through Nrf2 and its downstream targets. The novelty of this study lies in demonstrating the potential of OECs‐EXOs to enhance neurological function after TBI, thereby providing a new therapeutic approach and research direction for TBI neurorehabilitation. Furthermore, it demonstrates that the neuroprotective effects of OECs‐EXOs are closely related to the regulation of Nrf2 and ferroptosis pathways.

The mNSS score, together with the Morris water maze (MWM) and Barnes maze tests, provided comprehensive assessments of neurological function and cognitive deficits during TBI recovery in rats.^30^ In parallel, after TBI, astrocytes released S100β and neurons released NSE; these proteins are sensitive and easily detectable biomarkers that can be dynamically monitored and thus provide predictive value for assessing TBI severity.^31^ Moreover, H&E staining was used to assess brain tissue structure, whereas Nissl staining and NeuN protein levels were used to evaluate neuronal damage.^32^ In addition, caspase‐3 and Fas were measured as cell death‐related markers that reflect the activation of neuronal apoptosis after TBI and provide complementary molecular evidence of tissue and neuronal injury.^33^ Consistent with previous findings,^34^ the TBI group in this study exhibited significant motor deficits, impaired learning and memory, elevated levels of serum S100β and NSE, and pronounced necrosis, swelling, and vacuolation in the affected brain regions. Additionally, we observed neuronal disorganization, nuclear pyknosis, decreased neuronal cell counts, upregulation of the cell death–related genes caspase‐3 and Fas, and reduced Nrf2 levels. Notably, the EXO group showed significant improvement across all of these parameters. However, combining OECs‐EXOs with ML385 attenuated these therapeutic effects, suggesting that the underlying mechanism may be associated with Nrf2 regulation.

Furthermore, immunofluorescence analysis showed that DiD‐labeled OECs‐EXOs were taken up by neurons, astrocytes, and microglia after intranasal delivery. This suggests that OECs‐EXOs can interact with different cell types involved in TBI recovery. Notably, some OECs‐EXOs localized within NeuN+ neurons, suggesting a potential direct role in neuronal recovery. Uptake by astrocytes and microglia further supports the involvement of these glial cells in mediating the neuroinflammatory response and contributing to neuroprotection in the context of TBI. These findings highlight the potential of OECs‐EXOs to target both neuronal and glial cells in the brain, providing a multi‐pronged approach to enhancing recovery after TBI. Our findings are consistent with previous studies showing that exosomes can be taken up by neurons, astrocytes, and microglia, further supporting the role in modulating neuroinflammation and promoting recovery after injury.^35^, ^36^

The therapeutic mechanism of OECs‐EXOs may involve the inhibition of neuroinflammation via Nrf2 modulation. TBI‐induced cellular membrane disruption results in the release of damage‐associated molecular patterns (DAMPs), which trigger and amplify neuroinflammation.^37^ In response, levels of IL‐1β, IL‐6, and TNF‐α are rapidly upregulated, acting as early mediators of the post‐traumatic inflammatory response.^38^ In this study, OECs‐EXOs treatment significantly reduced the levels of IL‐1β, IL‐6, and TNF‐α. However, suppression of Nrf2 expression diminished these anti‐inflammatory effects. Supporting these findings, Soheila et al. reported that OECs reduced IL‐1β and TNF‐α levels, thereby alleviating neuroinflammation after spinal cord injury.^39^ Ailing et al. demonstrated that OECs activated the Nrf2 pathway, regulating neuroinflammatory factor levels and mitigating brain tissue injury.^26^ Taken together, Nrf2 may play a dual role in regulating neuroinflammation: it directly inhibits neurotoxic astrocyte activation by suppressing the recruitment of NF‐κB subunit p65,^7^ and it reduces pro‐inflammatory macrophage activation by inhibiting the recruitment of RNA polymerase II to the transcriptional start site of IL‐6 and IL‐1β, thereby downregulating the expression of these pro‐inflammatory genes.^9^

The therapeutic effects of OECs‐EXOs on TBI may also be closely linked to Nrf2‐mediated regulation of oxidative stress and ferroptosis. TBI‐induced oxidative stress disrupts the antioxidant system, leading to the accumulation of lipid peroxides and mitochondrial damage.^40^ Tissue hemorrhage results in iron overload, and excess Fe^2+^ engages in Fenton reactions to generate ROS, thereby exacerbating ferroptosis.^8^ In this study, OECs‐EXOs treatment significantly increased SOD activity and GSH levels while decreasing MDA and Fe^2+^ levels in brain tissue. Histopathological analysis revealed decreased Fe^2+^ accumulation and reduced mitochondrial damage, suggesting that OECs‐EXOs effectively ameliorate oxidative stress and iron imbalance. OECs enhance the antioxidant capacity of rat hippocampal tissue,^41^ and the combination of OECs with minocycline further reduces oxidative stress.^39^ OEC secretions have therapeutic potential in alleviating oxidative stress and mitochondrial dysfunction.^42^, ^43^ Furthermore, OECs can modulate oxidative stress markers and reduce cerebral underperfusion injury by activating the Nrf2 pathway.^26^ Nrf2 mitigates ROS accumulation by upregulating antioxidant genes and protects neurons and astrocytes from oxidative stress through the Nrf2‐antioxidant response element (ARE) signaling pathway.^15^ These studies support the current finding that OECs‐EXOs exert therapeutic effects in TBI by upregulating Nrf2, thereby inhibiting oxidative stress and ferroptosis.

Dodson et al. reported that many key proteins and enzymes that inhibit lipid peroxidation and ferroptosis are direct targets of Nrf2.^44^ Nrf2 also activates the expression of SLC7A11 and Gpx4 by binding to AREs in the promoter regions of these genes.^12^ SLC7A11 facilitates the uptake of amino acid substrates required for glutathione synthesis, while GPX4 inhibits ferroptosis by neutralizing lipid peroxides using glutathione.^10^ ACSL4, a marker of ferroptosis in TBI, increases cellular susceptibility to ferroptosis by regulating polyunsaturated fatty acid synthesis. It catalyzes the esterification of arachidonic acid‐CoA, generating large amounts of lipid ROS, which in turn induces ferroptosis.^45^ Nrf2 counteracts ACSL4‐induced lipid oxidation by upregulating antioxidant genes such as GPX4, thereby reducing lipid peroxidation. This indirect regulation is a crucial aspect of Nrf2's anti‐ferroptosis mechanism.^46^ Moreover, dysregulation of cellular iron metabolism is characterized by the upregulation of TFR1 and downregulation of ferritin. Excessive iron uptake coupled with ferritin deficiency leads to an increase in free iron, promoting lipid peroxidation and ROS generation and ultimately triggering ferroptosis.^8^ Under iron‐deficient conditions, Nrf2 upregulates TFR1 to enhance iron uptake, whereas under iron overload, Nrf2 promotes ferritin synthesis to store excess iron. This dual regulation enables Nrf2 to help cells adapt to different iron conditions, thereby preventing cellular damage caused by iron overload.

Change in Nrf2 and its regulatory targets, along with ferroptosis marker molecules were investigated following TBI. The results indicated that ferroptosis occurred post‐TBI, with downregulation of Nrf2, Gpx4, SLC7A11, and ferritin expression, as well as upregulation of ACSL4 and TFR1. OECs‐EXOs treatment significantly increased expression levels of Nrf2, Gpx4, SLC7A11, and ferritin, while decreasing those of ACSL4 and TFR1. However, with ML385 treatment, the expression of ferroptosis‐related molecules differed significantly from that in the EXO group, suggesting that Nrf2 may be a key mediator of the therapeutic effects of OECs‐EXOs in TBI. The anti‐inflammatory and antioxidant effects of irisin in sepsis‐associated encephalopathy are closely related to the Nrf2/Gpx4 axis, which regulates mitochondrial oxidative stress and ferroptosis; the expression patterns of Gpx4, SLC7A11, and ACSL4 in the present study were similar to these findings.^47^ As illustrated in the graphical abstract, we propose that the therapeutic mechanism of OECs‐EXOs in TBI may involve Nrf2‐mediated regulation of Gpx4, SLC7A11, ACSL4, TFR1, and ferritin. Modulation of these molecules triggers subsequent alterations in MDA, SOD, GSH, Fe^2+^ levels, as well as mitochondrial function, thereby affecting the ferroptosis pathway. This cascade of responses ultimately contributes to tissue and neuronal repair, reduces inflammation and oxidative stress, and improves neurological and cognitive function after TBI.

This study has several limitations, which also indicate directions for future work. First, although OECs‐EXOs showed therapeutic effects against TBI, the exact molecular identity and relative contribution of their active components remain unclear and require further investigation. Second, Nrf2 was assessed using pharmacological inhibition with ML385, and future work using Nrf2 knockout or overexpression models could provide more definitive evidence for its role. Third, the brain distribution of OECs‐EXOs was evaluated qualitatively by fluorescence imaging, but we did not determine the exact proportion of the administered dose accumulating in the brain or in specific regions; more quantitative imaging approaches will be helpful to better characterize their biodistribution and lesion targeting. Finally, only male rats were used in this study, which may limit the generalizability of the findings. Sex differences can influence immune responses and repair mechanisms, potentially leading to varied treatment outcomes.^48^ Studies have reported that females often have better outcomes in severe TBI models, possibly due to differences in hormonal influences or immune system function.^49^ Future studies should include both sexes to better understand sex‐specific effects of OECs‐EXOs. These limitations provide crucial directions for future research and will help improve the translational potential of OECs‐EXO‐based therapies.

## 4 METHODS

### 4.1 Animals

Sprague–Dawley (SD) rats were obtained from Fujian Medical University and housed at the Animal Center of Quanzhou University of Higher Medical Sciences. Before the experiments, rats were acclimatized for at least 1 week under controlled conditions (12 h light/dark cycle at 22 ± 0.5°C and 60 ± 10% humidity) with free access to food and water. Two‐week‐old male SD rats (~50 g) were used for OECs culture, whereas 7‐week‐old male rats (~250 g) were used for the TBI model. Rats assigned to the in vivo TBI experiments were randomized at the individual animal level into the Sham, TBI, EXO, and ML385 groups using a computer‐generated random number sequence with equal allocation, as described in section 4.4.2. All procedures were approved by the Ethics Committee of the Second Affiliated Hospital of Fujian Medical University (No. [2021] 256).

### 4.2 Culture and characterization of primary OECs

Primary OECs were cultured and purified from rat (~50 g) olfactory bulbs.^50^ The olfactory bulbs were obtained using Hank's 1× solution (Meilunbio, China), digested with 0.125% trypsin (Gibco, USA) for 25 min. The cell pellet was suspended in DMEM/F‐12 medium (Hyclone, USA) containing 20% fetal bovine serum (FBS, Gibco, USA), and 1% penicillin/ streptomycin (Meilunbio, China). The cell suspension was seeded into poly‐L‐lysine‐pretreated culture flasks, and OECs were purified using differential adhesion based on cell attachment times. OECs were identified by immunostaining with a p75 nerve growth factor receptor (p75NGFR) antibody (ET1601‐22, Huabio, China).

### 4.3 Isolation and identification of exosomes from OECs

#### 4.3.1 Exosomes isolation

When purified OECs reached approximately 80% confluence, the culture medium was replaced with FBS‐depleted medium. After 48 h, supernatants were collected and centrifuged at 2000*g* for 10 min and 10,000*g* for 30 min to remove cells and debris. The clarified supernatants were then ultracentrifuged at 100,000*g* for 70 min at 4°C to isolate exosomes, which were resuspended in phosphate‐buffered saline (PBS) and ultracentrifuged again at 100,000*g* for 70 min. The purified exosomes were finally resuspended in sterile PBS. For storage, exosome preparations were aliquoted (50–100 μL per tube) into low protein‐binding tubes, snap‐frozen on dry ice, and stored at −80°C. Each aliquot was subjected to no more than one freeze–thaw cycle. Thawing was performed on ice and preparations were used immediately after dilution (within 2 h).

#### 4.3.2 Exosomes identification

Exosome concentrations and size distributions were measured using a flow nanoanalyzer (nanoFCM, China). Exosomal markers CD9, CD81, and CD63 (555371, 551108, 556019, BD Biosciences, USA) were assessed using the same instrument. Exosomes were visualized by TEM (Hitachi, Japan). For batches intended for in vivo use within 4 weeks of preparation, exosome integrity was verified on a representative aliquot after storage and before administration using the methods described above. Preparations showing visible aggregation or a shift in modal size were discarded.

### 4.4 Manipulation and grouping of TBI rats

#### 4.4.1 Surgery on TBI rats

TBI was induced in rats using a controlled cortical impact (CCI) device (Zhongshi Dichuang, China). After anesthesia with 2% pentobarbital (50 mg/kg), a 3‐mm craniotomy was performed 2 mm posterior to the coronal suture and 2 mm lateral to the midline. A 2.5‐mm impactor tip delivered an impact at 5 m/s to a depth of 2 mm for 500 ms.^51^ After injury, the surgical site was disinfected, hemostasis was performed, and the scalp was sutured. In the Sham group, a craniotomy was performed without impact, followed by scalp suturing.

#### 4.4.2 TBI rats grouping

Rats were randomized at the individual animal level into the Sham, TBI, EXO (TBI + OECs‐EXOs), and ML385 (TBI + OECs‐EXOs + ML385) groups using a computer‐generated random number sequence with equal allocation (*n* = 20 per group). Outcome assessors were blinded to group assignments throughout data collection and analysis.

### 4.5 Nrf2 inhibition

The Nrf2 inhibitor, ML385 (MCE, USA), was dissolved in a vehicle consisting of 5% Tween80, 40% PEG300, 50% ddH2O, and 5% DMSO.^52^ Rats in the ML385 group received intraperitoneal injections of ML385 (30 mg/kg) once daily for 7 days, with TBI induced on Day 3.^53^ Equal volumes of the vehicle were administered to the Sham, TBI, and EXO groups as controls. The inhibitory effect of ML385 on Nrf2 was confirmed by western blot analysis.

### 4.6 OECs‐EXOs administration

#### 4.6.1 Dosage

**FIGURE 9:**
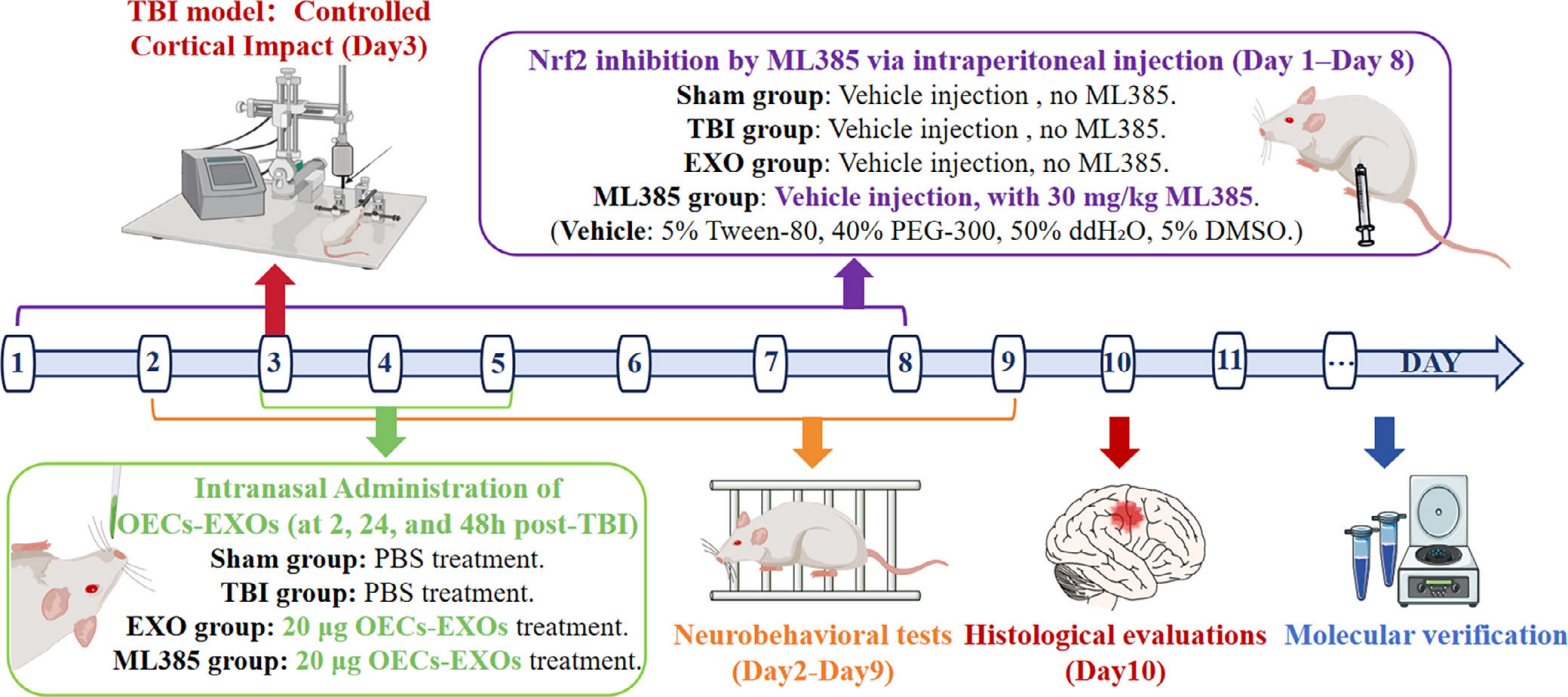
Experimental timeline for traumatic brain injury (TBI) induction and treatment.

Based on previous studies,^54^ the effective dose of OECs was 5 × 10^5^ cells per administration, corresponding to 20 μg of OECs‐EXOs (20 μL, 1 μg/μL). Treatments were administered at 2, 24, and 48 h post‐injury. EXO and ML385 groups received 20 μg of OECs‐EXOs, whereas the Sham and TBI groups received equal volumes of PBS (Figure 9).

#### 4.6.2 Intranasal administration

Rats were placed in a supine position under light anesthesia, and OECs‐EXOs were administered at a dose of 2.5 μL per drop, alternating nostrils every 2–3 min. The total administration time was 30 min, with a maximum volume of 25 μL.^55^ Post‐administration, rats remained in the supine position for 30 min to facilitate mucosal absorption.^56^

### 4.7 Tracking of administered OECs‐EXOs

To evaluate the feasibility of intranasal administration of OECs‐EXOs, the biodistribution and migration of DiR‐labeled OECs‐EXOs (Meilunbio, China) were monitored in vivo. The control group received an equal volume of PBS. Real‐time fluorescence imaging was performed using an AniView Kirin imaging system (Biolight Biotechnology, China) with 745 nm excitation and 820 nm emission filters to assess biodistribution at different time points. In addition, brain tissue sections were subjected to immunofluorescence staining to co‐localize DiD‐labeled OECs‐EXOs (Meilunbio, China) with neuronal (NeuN, ab177487, Abcam), astrocytic (GFAP, ab53554, Abcam), and microglial (Iba1, ab178846, Abcam) markers, thereby evaluating cellular uptake in the brain. Co‐localization analysis was performed to assess the interaction of OECs‐EXOs with different cell types in the brain following intranasal delivery.

### 4.8 Neurobehavioral tests

Neurobehavioral deficits were assessed by a blinded researcher using the mNSS. The mNSS is a comprehensive scale that evaluates motor, sensory, reflex, and balance functions. Scores of 13–18 indicated severe injury, 7–12 indicated moderate injury, and 1–6 indicated mild injury.^57^

### 4.9 Morris water maze test

The MWM test was used to evaluate spatial learning and memory.^58^ Rats were trained for 6 days with four acquisition trials per day to locate a hidden platform in opaque water. Escape latency served as the primary index of learning performance. On the seventh day, a probe test was performed after removal of the platform. The time between the platform in the target area and the detection track was used as an index. Behavioral data acquisition, analysis, and test recordings were performed using an automated tracking system and the Visu tracking system (Xinruan, China).

### 4.10 Barnes maze test

The Barnes maze test was used to reduce stress‐related bias associated with the MWM. Rats that did not undergo the MWM were tested in the Barnes maze to evaluate spatial learning and memory.^34^ The rats were habituated to the maze 1 week before acquisition training. During the acquisition training week, rats had 180 s to explore and escape, with guidance provided as needed. Training was performed for 6 days, with three trials per day. On Day 7, the escape box was removed for probe trials, and rats were observed for 90 s. The maze surface and escape box were cleaned with 75% ethanol after each trial. The maze platform was static, and the escape holes were identical. The trials were separated by 30 min.^59^

### 4.11 Histopathological evaluation

Brain samples were collected for histopathological analysis on Day 7 post‐TBI to evaluate the neuroprotective effects of OECs‐EXOs in TBI rats, with a focus on changes in brain tissue and neurons. Brain tissues were fixed in 4% paraformaldehyde, dehydrated, and embedded in paraffin. Processed samples were cut into 5 μm sections.

### 4.12 Staining protocol

The sections were deparaffinized, rehydrated, and stained with hematoxylin and eosin (H&E, Servicebio, China) and cresyl violet (Nissl, Servicebio, China). Prussian blue staining with DAB (Solarbio, China) was performed according to the manufacturer's instructions. Images were captured using a light microscope (Nikon, Tokyo, Japan).

### 4.13 Transmission electron microscopy

Brain samples from the lesion margins were post‐fixed in 2.5% phosphate‐buffered glutaraldehyde and 1% osmium tetroxide. Samples were cut and stained with 2% uranyl acetate, dehydrated through an acetone series, and embedded in epoxy resin. Sections (70–90 nm) were stained with lead citrate and uranyl acetate, and ultrastructural images were obtained using TEM (Hitachi, Japan).

### 4.14 Enzyme‐linked immunosorbent assay

Specific ELISA kits (Meimian, China) were used to analyze serum levels of interleukin (IL)‐1β, IL‐6, tumor necrosis factor‐alpha (TNF‐α), neuron‐specific enolase (NSE), and S100 calcium‐binding protein β (S100β). NSE and S100β serum levels were measured as indicators of the extent of brain damage.

### 4.15 Western blot

Proteins were extracted from injured brain tissues using RIPA buffer (Meilunbio, China), separated by SDS‐PAGE, and transferred to PVDF membranes (Millipore, USA). Membranes were blocked with 5% skimmed milk and incubated overnight at 4°C with primary antibodies: Gpx4 (ET1706‐45, Huabio), Acyl‐CoA synthetase long‐chain family member 4 (ACSL4, ET7111‐43, Huabio), Nrf2 (ER1706‐41, Huabio), SLC7A11 (HA721868, Huabio), transferrin receptor 1 (TFR1, ET1702‐06, Huabio), ferritin (ET1610‐78, Huabio), neuron‐specific nuclear protein (NeuN, ET1602‐12, Huabio), and GAPDH (ET1601‐4, Huabio). Horseradish peroxidase‐conjugated secondary antibodies were applied, and signals were detected using ECL reagents (Solarbio, China) and an imaging system (GE Healthcare, USA). Quantification was performed using ImageJ software.

### 4.16 Assessment of malondialdehyde, glutathione, superoxide dismutase, and Fe^2+^

The levels of malondialdehyde (MDA), reduced GSH, superoxide dismutase (SOD), and iron in the injured brain tissue were evaluated using assay kits provided by Elabscience Biotechnology Co., Ltd. (China).

### 4.17 Quantitative real‐time polymerase chain reaction analysis

Total RNA was extracted using an RNA extraction kit (Beyotime, China). Reverse transcription was performed using the Revert Aid First‐Strand cDNA Synthesis Kit (Toyobo, Japan). Gene expression levels were normalized to glyceraldehyde phosphate dehydrogenase (GAPDH). The primer sequences are listed in Table 1.

**TABLE 1:** qPCR primer sequences.

Name	Forward (5′–3)	Reverse (5′–3)
TNF‐α	CCGAGATGTGGAACTGGCAGAG	CCACGAGCAGGAATGAGAAGAGG
IL‐1β	TCTCACAGCAGCATCTCGACAAG	CCACGGGCAAGACATAGGTAGC
IL‐6	GCCTTCTTGGGACTGATGTTGTTG	GTCTGTTGTGGGTGGTATCCTCTG
ACSL4	CCATATCGCTCTGTCACGCACTTC	CCAGGCTGTCCTTCTTCCCAAAC
β‐Actin	ACTATCGGCAATGAGCGGTTCC	TGGCATAGAGGTCTTTACGGATGTC
Nrf2	ATGCCTTCCTCTGCTGCCATTAG	ACCGTGCCTTCAGTGTGCTTC
TFR1	TCAGCAAAGTCTGGCGAGATGAAC	GAGCCTCCACTGGGTCAATGTTAC
Ferritin	TGCCATCAACCGCCAGATCAAC	AAGTTCTTCAGGGCCACATCATCC
Gpx4	AGCCCATTCCCGAGCCTTTC	GATGCACACAAGCCCAGGAAC
SLC7A11	TCATCATCGGCACCGTCATCG	CTCCACAGGCAGACCAGAACAC
GAPDH	AAGTTCAACGGCACAGTCAAGG	GACATACTCAGCACCAGCATCAC
Caspase‐3	ACTACTGCCGGAGTCTGACT	TAACCGGGTGCGGTAGAGTA
Fas	ACATCCTTGAGCCTTGCACA	ATCAGCAGCCAAAGGAGCTT

### 4.18 Statistical analysis

Statistical analyses were performed using ImageJ software (National Institutes of Health, Bethesda, MD, USA) and GraphPad Prism 9.5 (San Diego, CA, USA). Normally distributed data were analyzed using one‐way analysis of variance (ANOVA) and least significant difference (LSD) post hoc tests. Motor and behavioral scores were analyzed using two‐way repeated measures ANOVA with Tukey's test. Sphericity corrections or multivariate ANOVA were applied when necessary. Significance was set at *p* < 0.05, and data were presented as mean ± *SEM*.

## 5 CONCLUSION

OECs‐EXOs regulate ferroptosis by modulating the levels of key molecules, such as Gpx4, SLC7A11, ferritin, TFR1, and ACSL4, via the Nrf2 signaling pathway. This regulation subsequently inhibits neuroinflammation and oxidative stress, decreases Fe^2+^ accumulation in the cerebral cortex, attenuates mitochondrial damage, and improves neurological and cognitive function in TBI rats, thereby exerting therapeutic effects in TBI. These findings provide a theoretical foundation for considering OECs‐EXOs as an alternative to OECs in TBI treatment and offer valuable new targets and insights for future research in TBI therapy.

## AUTHOR CONTRIBUTIONS

**Yi‐bin Liu**: Conceived the study. **Xin‐li Chen**: Conducted experiments and drafted the manuscript. **Cheng‐ye Lin**: Analyzed data. **Shu Lin**: Edited the manuscript. **He‐fan He**: Funding acquisition; writing – review editing; resources; formal analysis. **Wei‐feng Liu**: Funding acquisition; project administration; supervision.

## CONFLICT OF INTEREST STATEMENT

The authors declare no conflicts of interest.

## Data Availability

The data that support the findings of this study are available from the corresponding author upon reasonable request.

## References

[1] Maas AIR , Menon DK , Adelson PD , et al. Traumatic brain injury: integrated approaches to improve prevention, clinical care, and research. Lancet Neurol. 2017;16(12):987‐1048. doi:10.1016/S1474-4422(17)30371-X 29122524

[2] Jiang J‐Y , Gao G‐Y , Feng J‐F , et al. Traumatic brain injury in China. Lancet Neurol. 2019;18(3):286‐295. doi:10.1016/S1474-4422(18)30469-1 30784557

[3] Kaur P , Sharma S . Recent advances in pathophysiology of traumatic brain injury. Curr Neuropharmacol. 2018;16(8):1224‐1238. doi:10.2174/1570159X15666170613083606 28606040 PMC6142406

[4] Xie B‐S , Wang Y‐Q , Lin Y , et al. Inhibition of ferroptosis attenuates tissue damage and improves long‐term outcomes after traumatic brain injury in mice. CNS Neurosci Ther. 2019;25(4):465‐475. doi:10.1111/cns.13069 30264934 PMC6488926

[5] Kenny EM , Fidan E , Yang Q , et al. Ferroptosis contributes to neuronal death and functional outcome after traumatic brain injury. Crit Care Med. 2019;47(3):410‐418. doi:10.1097/CCM.0000000000003555 30531185 PMC6449247

[6] Hu X , Xu Y , Xu H , et al. Progress in understanding ferroptosis and its targeting for therapeutic benefits in traumatic brain and spinal cord injuries. Front Cell Dev Biol. 2021;9:705786. doi:10.3389/fcell.2021.705786 34422826 PMC8371332

[7] Wenzel SE , Tyurina YY , Zhao J , et al. PEBP1 wardens ferroptosis by enabling lipoxygenase generation of lipid death signals. Cell. 2017;171(3):628‐641.e26. doi:10.1016/j.cell.2017.09.044 29053969 PMC5683852

[8] Stockwell BR . Ferroptosis turns 10: emerging mechanisms, physiological functions, and therapeutic applications. Cell. 2022;185(14):2401‐2421. doi:10.1016/j.cell.2022.06.003 35803244 PMC9273022

[9] Sun X , Ou Z , Chen R , et al. Activation of the p62‐Keap1‐NRF2 pathway protects against ferroptosis in hepatocellular carcinoma cells. Hepatology. 2016;63(1):173‐184. doi:10.1002/hep.28251 26403645 PMC4688087

[10] Deng H‐F , Yue L‐X , Wang N‐N , et al. Mitochondrial iron overload‐mediated inhibition of Nrf2‐HO‐1/GPX4 assisted ALI‐induced nephrotoxicity. Front Pharmacol. 2020;11:624529. doi:10.3389/fphar.2020.624529 33584308 PMC7873870

[11] Kerins MJ , Ooi A . The roles of NRF2 in modulating cellular iron homeostasis. Antioxid Redox Signal. 2018;29(17):1756‐1773. doi:10.1089/ars.2017.7176 28793787 PMC6208163

[12] Zhao T , Yu Z , Zhou L , et al. Regulating Nrf2‐GPx4 axis by bicyclol can prevent ferroptosis in carbon tetrachloride‐induced acute liver injury in mice. Cell Death Discov. 2022;8(1):380. doi:10.1038/s41420-022-01173-4 36071041 PMC9452542

[13] Lane DJR , Metselaar B , Greenough M , Bush AI , Ayton SJ . Ferroptosis and NRF2: an emerging battlefield in the neurodegeneration of Alzheimer's disease. Essays Biochem. 2021;65(7):925‐940. doi:10.1042/EBC20210017 34623415

[14] Assinck P , Duncan GJ , Hilton BJ , Plemel JR , Tetzlaff W . Cell transplantation therapy for spinal cord injury. Nat Neurosci. 2017;20(5):637‐647. doi:10.1038/nn.4541 28440805

[15] Roet KCD , Verhaagen J . Understanding the neural repair‐promoting properties of olfactory ensheathing cells. Exp Neurol. 2014;261:594‐609. doi:10.1016/j.expneurol.2014.05.007 24842489

[16] Lindsay SL , McCanney GA , Willison AG , Barnett SC . Multi‐target approaches to CNS repair: olfactory mucosa‐derived cells and heparan sulfates. Nat Rev Neurol. 2020;16(4):229‐240. doi:10.1038/s41582-020-0311-0 32099190

[17] Ziegler MD , Hsu D , Takeoka A , et al. Further evidence of olfactory ensheathing glia facilitating axonal regeneration after a complete spinal cord transection. Exp Neurol. 2011;229(1):109‐119. doi:10.1016/j.expneurol.2011.01.007 21272578 PMC3085566

[18] Iwatsuki K , Tajima F , Ohnishi Y‐I , et al. A pilot clinical study of olfactory mucosa autograft for chronic complete spinal cord injury. Neurol Med Chir (Tokyo). 2016;56(6):285‐292. doi:10.2176/nmc.oa.2015-0320 27053327 PMC4908071

[19] Dlouhy BJ , Awe O , Rao RC , Kirby PA , Hitchon PW . Autograft‐derived spinal cord mass following olfactory mucosal cell transplantation in a spinal cord injury patient: case report. J Neurosurg Spine. 2014;21(4):618‐622. doi:10.3171/2014.5.SPINE13992 25002238

[20] Zhang L‐P , Liao J‐X , Liu Y‐Y , Luo H‐L , Zhang W‐J . Potential therapeutic effect of olfactory ensheathing cells in neurological diseases: neurodegenerative diseases and peripheral nerve injuries. Front Immunol. 2023;14:1280186. doi:10.3389/fimmu.2023.1280186 37915589 PMC10616525

[21] Tu Y‐K , Hsueh Y‐H . Extracellular vesicles isolated from human olfactory ensheathing cells enhance the viability of neural progenitor cells. Neurol Res. 2020;42(11):959‐967. doi:10.1080/01616412.2020.1794371 32700620

[22] Yang B , Chen Y , Shi J . Exosome biochemistry and advanced nanotechnology for next‐generation theranostic platforms. Adv Mater. 2019;31(2):e1802896. doi:10.1002/adma.201802896 30126052

[23] Zhang Y , Chopp M , Meng Y , et al. Effect of exosomes derived from multipluripotent mesenchymal stromal cells on functional recovery and neurovascular plasticity in rats after traumatic brain injury. J Neurosurg. 2015;122(4):856‐867. doi:10.3171/2014.11.JNS14770 25594326 PMC4382456

[24] Sharma P , Mesci P , Carromeu C , et al. Exosomes regulate neurogenesis and circuit assembly. Proc Natl Acad Sci U S A. 2019;116(32):16086‐16094. doi:10.1073/pnas.1902513116 31320591 PMC6689941

[25] Fan H , Chen Z , Tang H‐B , et al. Exosomes derived from olfactory ensheathing cells provided neuroprotection for spinal cord injury by switching the phenotype of macrophages/microglia. Bioeng Transl Med. 2022;7(2):e10287. doi:10.1002/btm2.10287 35600663 PMC9115713

[26] Yu A , Mao L , Zhao F , Sun B . Olfactory ensheathing cells transplantation attenuates chronic cerebral hypoperfusion induced cognitive dysfunction and brain damages by activating Nrf2/HO‐1 signaling pathway. Am J Transl Res. 2018;10(10):3111‐3121. 30416654 PMC6220231

[27] Théry C , Witwer KW , Aikawa E , et al. Minimal information for studies of extracellular vesicles 2018 (MISEV2018): a position statement of the International Society for Extracellular Vesicles and update of the MISEV2014 guidelines. J Extracell Vesicles. 2018;7(1):1535750. doi:10.1080/20013078.2018.1535750 30637094 PMC6322352

[28] Ghosh S , Garg S , Ghosh S . Cell‐derived exosome therapy: a novel approach to treat post‐traumatic brain injury mediated neural injury. ACS Chem Nerosci. 2020;11(14):2045‐2047. doi:10.1021/acschemneuro.0c00368 32609493

[29] Cheng H , Wang P , Wang N , et al. Neuroprotection of NRF2 against ferroptosis after traumatic brain injury in mice. Antioxidants (Basel). 2023;12(3):731. doi:10.3390/antiox12030731 36978979 PMC10044792

[30] Shultz SR , McDonald SJ , Corrigan F , et al. Clinical relevance of behavior testing in animal models of traumatic brain injury. J Neurotrauma. 2020;37(22):2381‐2400. doi:10.1089/neu.2018.6149 30907237

[31] Wang KK , Yang Z , Zhu T , et al. An update on diagnostic and prognostic biomarkers for traumatic brain injury. Expert Rev Mol Diagn. 2018;18(2):165‐180. doi:10.1080/14737159.2018.1428089 29338452 PMC6359936

[32] Xu X , Gao W , Cheng S , et al. Anti‐inflammatory and immunomodulatory mechanisms of atorvastatin in a murine model of traumatic brain injury. J Neuroinflammation. 2017;14(1):167. doi:10.1186/s12974-017-0934-2 28835272 PMC5569493

[33] Yang Y , Lu D , Wang M , et al. Endoplasmic reticulum stress and the unfolded protein response: emerging regulators in progression of traumatic brain injury. Cell Death Dis. 2024;15(2):156. doi:10.1038/s41419-024-06515-x 38378666 PMC10879178

[34] Fesharaki‐Zadeh A , Miyauchi JT , St Laurent‐Arriot K , Tsirka SE , Bergold PJ . Increased behavioral deficits and inflammation in a mouse model of co‐morbid traumatic brain injury and post‐traumatic stress disorder. ASN Neuro. 2020;12:1759091420979567. doi:10.1177/1759091420979567 33342261 PMC7755938

[35] Pantazopoulou M , Lamprokostopoulou A , Karampela DS , et al. Differential intracellular trafficking of extracellular vesicles in microglia and astrocytes. Cell Mol Life Sci. 2023;80(7):193. doi:10.1007/s00018-023-04841-5 37391572 PMC10313565

[36] Men Y , Yelick J , Jin S , et al. Exosome reporter mice reveal the involvement of exosomes in mediating neuron to astroglia communication in the CNS. Nat Commun. 2019;10(1):4136. doi:10.1038/s41467-019-11534-w 31515491 PMC6742670

[37] Corps KN , Roth TL , McGavern DB . Inflammation and neuroprotection in traumatic brain injury. JAMA Neurol. 2015;72(3):355‐362. doi:10.1001/jamaneurol.2014.3558 25599342 PMC5001842

[38] Ransohoff RM , Brown MA . Innate immunity in the central nervous system. J Clin Invest. 2012;122(4):1164‐1171. doi:10.1172/JCI58644 22466658 PMC3314450

[39] Pourkhodadad S , Oryan SH , Kaka G , Sadraie SH . Neuroprotective effects of combined treatment with minocycline and olfactory ensheathing cells transplantation against inflammation and oxidative stress after spinal cord injury. Cell J. 2019;21(2):220‐228. doi:10.22074/cellj.2019.6126 30825296 PMC6397610

[40] Fesharaki‐Zadeh A . Oxidative stress in traumatic brain injury. Int J Mol Sci. 2022;23(21):13000. doi:10.3390/ijms232113000 36361792 PMC9657447

[41] Liu W , Zheng Q , Wang Y , Han X , Yuan L , Zhao M . Transplantation of olfactory ensheathing cells attenuates acute carbon monoxide poisoning‐induced brain damages in rats. Neurochem Res. 2015;40(1):70‐80. doi:10.1007/s11064-014-1467-z 25370793

[42] Shukla A , Mohapatra TM , Parmar D , Seth K . Neuroprotective potentials of neurotrophin rich olfactory ensheathing cell's conditioned media against 6OHDA‐induced oxidative damage. Free Radic Res. 2014;48(5):560‐571. doi:10.3109/10715762.2014.894636 24528157

[43] Fu Q‐Q , Wei L , Sierra J , et al. Olfactory ensheathing cell‐conditioned medium reverts Aβ25‐35‐induced oxidative damage in SH‐SY5Y cells by modulating the mitochondria‐mediated apoptotic pathway. Cell Mol Neurobiol. 2017;37(6):1043‐1054. doi:10.1007/s10571-016-0437-1 27807758 PMC11482107

[44] Dodson M , Castro‐Portuguez R , Zhang DD . NRF2 plays a critical role in mitigating lipid peroxidation and ferroptosis. Redox Biol. 2019;23:101107. doi:10.1016/j.redox.2019.101107 30692038 PMC6859567

[45] Wang H , Liu C , Zhao Y , Gao G . Mitochondria regulation in ferroptosis. Eur J Cell Biol. 2020;99(1):151058. doi:10.1016/j.ejcb.2019.151058 31810634

[46] Hsu YL , Hung JY , Chang WA , et al. Hypoxic lung cancer‐secreted exosomal miR‐23a increased angiogenesis and vascular permeability by targeting prolyl hydroxylase and tight junction protein ZO‐1. Oncogene. 2017;36(34):4929‐4942. doi:10.1038/onc.2017.105 28436951

[47] Wang J , Zhu Q , Wang Y , Peng J , Shao L , Li X . Irisin protects against sepsis‐associated encephalopathy by suppressing ferroptosis via activation of the Nrf2/GPX4 signal axis. Free Radic Biol Med. 2022;187:171‐184. doi:10.1016/j.freeradbiomed.2022.05.023 35660523

[48] Jha RM , Rajasundaram D , Sneiderman C , et al. A single‐cell atlas deconstructs heterogeneity across multiple models in murine traumatic brain injury and identifies novel cell‐specific targets. Neuron. 2024;112(18):3069‐3088.e4. doi:10.1016/j.neuron.2024.06.021 39019041 PMC11578855

[49] Svedung Wettervik T , Hånell A , Howells T , Enblad P , Lewén A . Females exhibit better cerebral pressure autoregulation, less mitochondrial dysfunction, and reduced excitotoxicity after severe traumatic brain injury. J Neurotrauma. 2022;39(21–22):1507‐1517. doi:10.1089/neu.2022.0097 35587145

[50] Reshamwala R , Shah M , Belt L , Ekberg JAK , St John JA . Reliable cell purification and determination of cell purity: crucial aspects of olfactory ensheathing cell transplantation for spinal cord repair. Neural Regen Res. 2020;15(11):2016‐2026. doi:10.4103/1673-5374.282218 32394949 PMC7716040

[51] Ma X , Aravind A , Pfister BJ , Chandra N , Haorah J . Animal models of traumatic brain injury and assessment of injury severity. Mol Neurobiol. 2019;56(8):5332‐5345. doi:10.1007/s12035-018-1454-5 30603958

[52] Duan C , Jiao D , Wang H , et al. Activation of the PPARγ prevents ferroptosis‐induced neuronal loss in response to intracerebral hemorrhage through synergistic actions with the Nrf2. Front Pharmacol. 2022;13:869300. doi:10.3389/fphar.2022.869300 35517804 PMC9065416

[53] Xian P , Hei Y , Wang R , et al. Mesenchymal stem cell‐derived exosomes as a nanotherapeutic agent for amelioration of inflammation‐induced astrocyte alterations in mice. Theranostics. 2019;9(20):5956‐5975. doi:10.7150/thno.33872 31534531 PMC6735367

[54] Li H , Yin Z , Yue S , et al. Effect of valproic acid combined with transplantation of olfactory ensheathing cells modified by neurotrophic 3 gene on nerve protection and repair after traumatic brain injury. Neuropeptides. 2024;103:102389. doi:10.1016/j.npep.2023.102389 37945445

[55] Kodali M , Castro OW , Kim D‐K , et al. Intranasally administered human MSC‐derived extracellular vesicles pervasively incorporate into neurons and microglia in both intact and status epilepticus injured forebrain. Int J Mol Sci. 2019;21(1):181. doi:10.3390/ijms21010181 31888012 PMC6981466

[56] Wang Y , Niu H , Li L , et al. Anti‐CHAC1 exosomes for nose‐to‐brain delivery of miR‐760‐3p in cerebral ischemia/reperfusion injury mice inhibiting neuron ferroptosis. J Nanobiotechnol. 2023;21(1):109. doi:10.1186/s12951-023-01862-x PMC10041751 36967397

[57] Hernandes MS , D'Avila JC , Trevelin SC , et al. The role of Nox2‐derived ROS in the development of cognitive impairment after sepsis. J Neuroinflammation. 2014;11:36. doi:10.1186/1742-2094-11-36 24571599 PMC3974031

[58] Li N , Wang W , Zhou H , et al. Ferritinophagy‐mediated ferroptosis is involved in sepsis‐induced cardiac injury. Free Radic Biol Med. 2020;160:303‐318. doi:10.1016/j.freeradbiomed.2020.08.009 32846217

[59] Gee CC , Steffen R , Kievit FM . An updated Barnes maze protocol for assessing the outcome of controlled cortical impact mouse models of traumatic brain injury. J Neurosci Methods. 2023;392:109866. doi:10.1016/j.jneumeth.2023.109866 37116622 PMC10205663

